# Author Correction: Environmental variability supports chimpanzee behavioural diversity

**DOI:** 10.1038/s41467-021-21010-z

**Published:** 2021-01-25

**Authors:** Ammie K. Kalan, Lars Kulik, Mimi Arandjelovic, Christophe Boesch, Fabian Haas, Paula Dieguez, Christopher D. Barratt, Ekwoge E. Abwe, Anthony Agbor, Samuel Angedakin, Floris Aubert, Emmanuel Ayuk Ayimisin, Emma Bailey, Mattia Bessone, Gregory Brazzola, Valentine Ebua Buh, Rebecca Chancellor, Heather Cohen, Charlotte Coupland, Bryan Curran, Emmanuel Danquah, Tobias Deschner, Dervla Dowd, Manasseh Eno-Nku, J. Michael Fay, Annemarie Goedmakers, Anne-Céline Granjon, Josephine Head, Daniela Hedwig, Veerle Hermans, Kathryn J. Jeffery, Sorrel Jones, Jessica Junker, Parag Kadam, Mohamed Kambi, Ivonne Kienast, Deo Kujirakwinja, Kevin E. Langergraber, Juan Lapuente, Bradley Larson, Kevin C. Lee, Vera Leinert, Manuel Llana, Sergio Marrocoli, Amelia C. Meier, Bethan Morgan, David Morgan, Emily Neil, Sonia Nicholl, Emmanuelle Normand, Lucy Jayne Ormsby, Liliana Pacheco, Alex Piel, Jodie Preece, Martha M. Robbins, Aaron Rundus, Crickette Sanz, Volker Sommer, Fiona Stewart, Nikki Tagg, Claudio Tennie, Virginie Vergnes, Adam Welsh, Erin G. Wessling, Jacob Willie, Roman M. Wittig, Yisa Ginath Yuh, Klaus Zuberbühler, Hjalmar S. Kühl

**Affiliations:** 1 Max Planck Institute for Evolutionary Anthropology, 04103 Leipzig, Germany; 2 Wild Chimpanzee Foundation, 04103 Leipzig, Germany; 3 German Centre for Integrative Biodiversity Research (iDiv) Halle-Jena-Leipzig, Leipzig, 04103 Germany; 4 Ebo Forest Research Project, BP3055 Messa, Cameroon; 5 Institute for Conservation Research, San Diego Zoo Global, Escondido, CA 92027-7000 USA; 6 West Chester University, Department of Psychology, West Chester, PA 19382 USA; 7 West Chester University, Department of Anthropology and Sociology, West Chester, PA 19382 USA; 8 Wildlife Conservation Society, New York, NY 10460 USA; 9 Department of Wildlife and Range Management, Faculty of Renewable Natural Resources, Kwame Nkrumah University of Science and Technology, Kumasi, Ghana; 10 WWF Cameroon Country Programme Office, BP 6776 Yaoundé, Cameroon; 11 Wonga-Wongue Reserve, Libreville, Gabon; 12 Chimbo Foundation, 1011 PW Amsterdam, Netherlands; 13 Elephant Listening Project, Bioacoustics Research Program, Cornell Lab of Ornithology, Cornell University, Ithaca, NY 14850 USA; 14 KMDA, Centre for Research and Conservation, Royal Zoological Society of Antwerp, B-2018 Antwerp, Belgium; 15 School of Natural Sciences, University of Stirling, Stirling, UK; 16 Agence National des Parcs Nationaux, BP20379 Libreville, Gabon; 17 University of Cambridge, Cambridge, UK CB2 3QG; 18 School of Human Evolution and Social Change & Institute of Human Origins, Arizona State University, Tempe, AZ 85287 USA; 19 Comoé Chimpanzee Conservation Project, Würzburg, Germany; 20 Jane Goodall Institute Spain and Senegal, Dindefelo Biological Station, Dindefelo, Kedougou Senegal; 21 Lester E. Fisher Center for the Study and Conservation of Apes, Lincoln Park Zoo, Chicago, IL 60614 USA; 22 Wildlife Conservation Society, Congo Program, B.P. 14537 Brazzaville, Republic of Congo; 23 Born Free Foundation, Broadlands Business Campus, Horsham, RH12 4QP UK; 24 School of Natural Sciences and Psychology, Liverpool John Moores University, James Parsons Building, Liverpool, L3 3AF UK; 25 University College London, Department of Anthropology, London, WC1H 0BW UK; 26 Washington University in Saint Louis, Department of Anthropology, St. Louis, MO 63130 USA; 27 Kyoto University Institute for Advanced Study, Kyoto University, Yoshida-Ushinomiya-cho, Sakyo-Ku, Kyoto, 606-8501 Japan; 28 Gashaka Primate Project, Serti, Taraba, Nigeria; 29 Department for Early Prehistory and Quaternary Ecology, University of Tübingen, 72070 Tübingen, Germany; 30 Department of Human Evolutionary Biology, Harvard University, Cambridge, MA 02138 USA; 31 Taï Chimpanzee Project, CSRS, Abidjan, Côte d’Ivoire; 32 Université de Neuchâtel, Institut de Biologie, 2000 Neuchâtel, Switzerland; 33 School of Psychology and Neuroscience, University of St Andrews, St Andrews, UK

Correction to: *Nature Communications* 10.1038/s41467-020-18176-3, published online 15 September 2020.

The original version of the Supplementary Information associated with this Article included an incorrect Supplementary Data 1 file, in which three columns (L, M and P) had slightly different variable names from those written in the code. The HTML has been updated to include a corrected version of Supplementary Data 1; the correct version of Supplementary Data 1 can be found as Supplementary Information associated with this Correction.

## Supplementary information

Supplementary Data 1

