# Supplementary material for: Environmental variability supports chimpanzee behavioural diversity
Source: Nat Commun. 2020 Sep 15;11:4451. doi: 10.1038/s41467-020-18176-3 (PMC7493986; doi:10.1038/s41467-020-18176-3)
Supplement: Supplementary file 1 — Supplementary Information [file 41467_2020_18176_MOESM1_ESM.pdf]

Supplementary Information

Environmental variability supports chimpanzee behavioural diversity

Kalan et al.

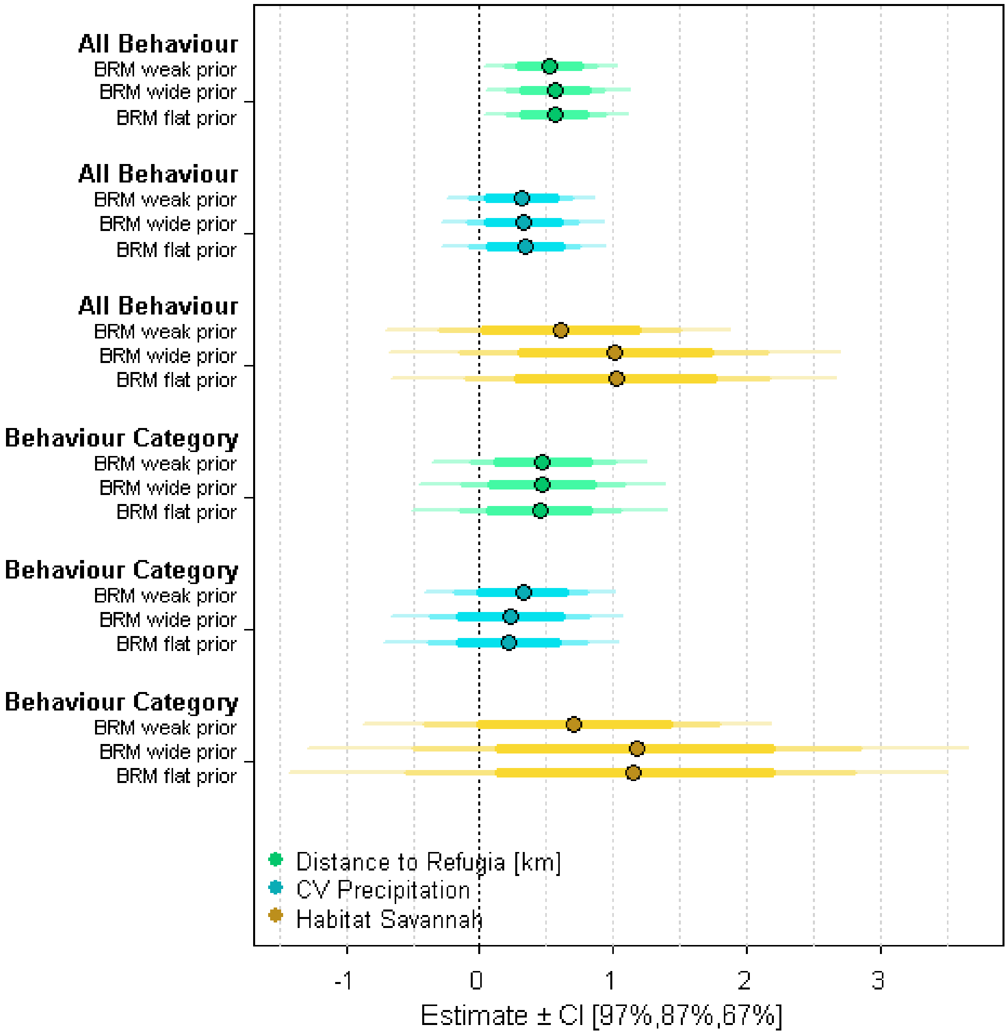

<u>Supplementary Figure 1.</u> **Prior sensitivity analysis demonstrating variation in effects of the three environmental variability predictors on the probability of occurrence for 31 chimpanzee behaviours and for 13 behavioural categories according to the prior chosen.** We present the weak prior results in the main text and for all variables. As expected, credible intervals are wider when using wide or flat priors while estimates vary minimally. The plot shows the mean of the marginal posterior distribution (dots) and the 67%, 87%, 97% credible intervals centred on the mean (coloured areas) for n=144 chimpanzee communities.

<u>Supplementary Table 1.</u> Results of Bayesian Regression Models for alternative response variables of behavioural categories, tool use behaviours only and non-tool use behaviours only using weak priors and testing environmental variability (EV) predictors one at a time. Estimate of the mean marginal posterior distribution, standard deviation of the estimate (sd), the 2.5% and 97.5% credible intervals centred on the mean (CI) and proportion of the posterior distribution greater than zero are given.

		Estimate	sd	CI 2.5%	CI 97.5%	Post. Dist. >0
**Behavioural categories (n=144)**						
	Intercept*	-3.43	0.82	-5.135	-1.888	-
EV predictors	distance to refugia	0.473	0.359	-0.262	1.156	0.913
	CV precipitation	0.331	0.327	-0.337	0.930	0.840
	habitat_savanna	0.710	0.723	-0.729	2.070	0.838
Control Predictors*	human footprint	-0.256	0.190	-0.638	0.112	0.083
	observation months	0.949	0.364	0.198	1.645	0.991
	ssp_*ellioti*	-0.280	0.754	-1.776	1.174	0.362
	ssp_*schweinfurthii*	-0.460	0.669	-1.772	0.837	0.243
	ssp_*troglodytes*	-0.347	0.751	-1.851	1.084	0.326
**Tool use behaviours (n=144)**						
	Intercept*	-4.354	0.606	-5.608	-3.217	-
EV predictors	distance to refugia	0.374	0.286	-0.189	0.930	0.908
	CV precipitation	0.216	0.309	-0.421	0.817	0.769
	habitat_savanna	0.311	0.669	-1.083	1.586	0.688
Control Predictors*	human footprint	-0.250	0.183	-0.622	0.104	0.082
	observation months	0.886	0.306	0.276	1.480	0.997
	ssp_ellioti	-0.014	0.753	-1.568	1.391	0.505
	ssp_schweinfurthii	-0.599	0.627	-1.837	0.611	0.166
	ssp_troglodytes	-0.223	0.742	-1.708	1.196	0.389
**Non-tool use behaviours (n=144)**						
	Intercept*	-5.202	1.046	-7.374	-3.347	-
EV predictors	distance to refugia	0.744	0.442	-0.141	1.614	0.951
	CV precipitation	0.219	0.506	-0.849	1.188	0.691
	habitat_savanna	0.017	0.881	-1.736	1.706	0.518
Control Predictors*	human footprint	-0.478	0.325	-1.151	0.143	0.064
	observation months	0.851	0.525	-0.236	1.826	0.943
	ssp_ellioti	-0.510	0.867	-2.215	1.189	0.273
	ssp_schweinfurthii	-0.166	0.798	-1.751	1.387	0.422
	ssp_troglodytes	0.077	0.788	-1.493	1.602	0.544

*averaged across all three EV models per response

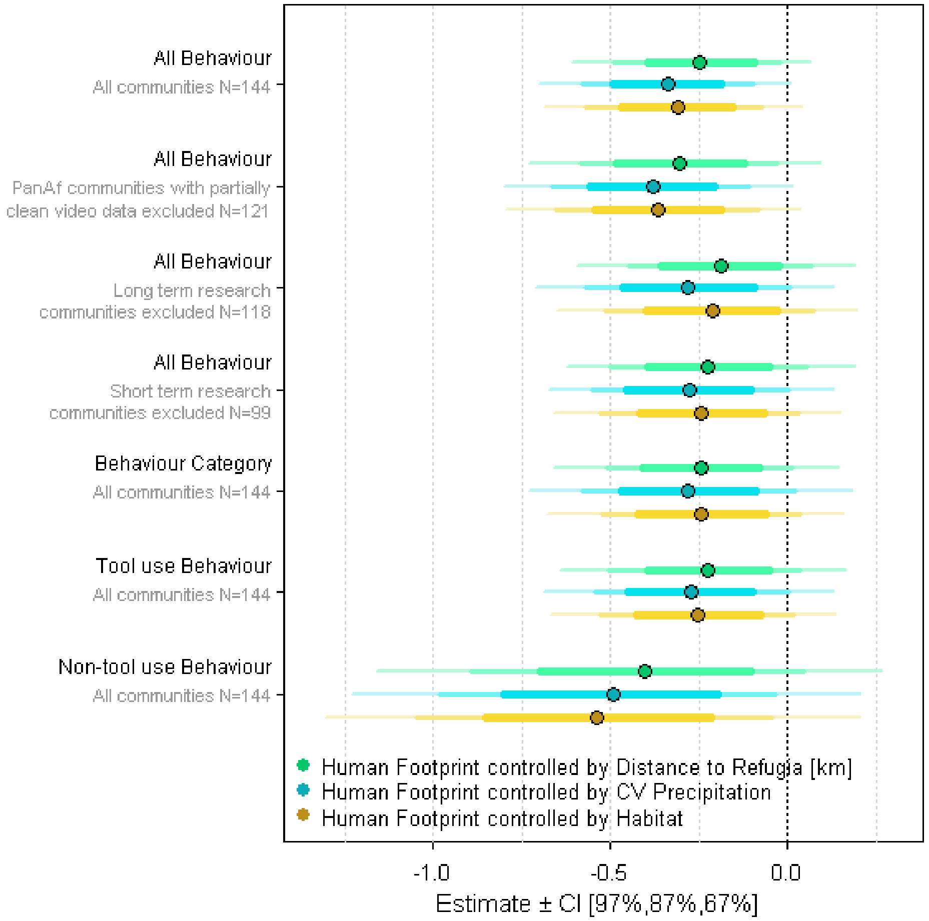

<u>**Supplementary Figure 2.**</u> **The negative influence of the control variable human footprint using a weak prior is evident in all models testing the effects of three environmental variability predictors.** The plot shows the mean of the marginal posterior distribution (dots) and the 67%, 87%, 97% credible intervals centred on the mean (coloured areas).

__Supplementary Table 2.__ **Results of the Gaussian Process used to control for spatial autocorrelation for the all behaviour model using weak priors and testing environmental variability predictors one at a time (n=144).** Estimate of the mean marginal posterior distribution, standard deviation of the estimate (Sd), and the 2.5% and 97.5% credible intervals centred on the mean (CI).

		Estimate	Sd	CI 2.5%	CI 97.5%
distance to refugia	sdgp(gplonglat)	0.705	0.169	0.367	1.025
	lscale(gplonglat)	0.001	0.001	0.000	0.003
CV precipitation	sdgp(gplonglat)	0.792	0.175	0.444	1.140
	lscale(gplonglat)	0.001	0.004	0.000	0.008
habitat_savanna	sdgp(gplonglat)	0.855	0.162	0.539	1.180
	lscale(gplonglat)	0.001	0.001	0.000	0.003

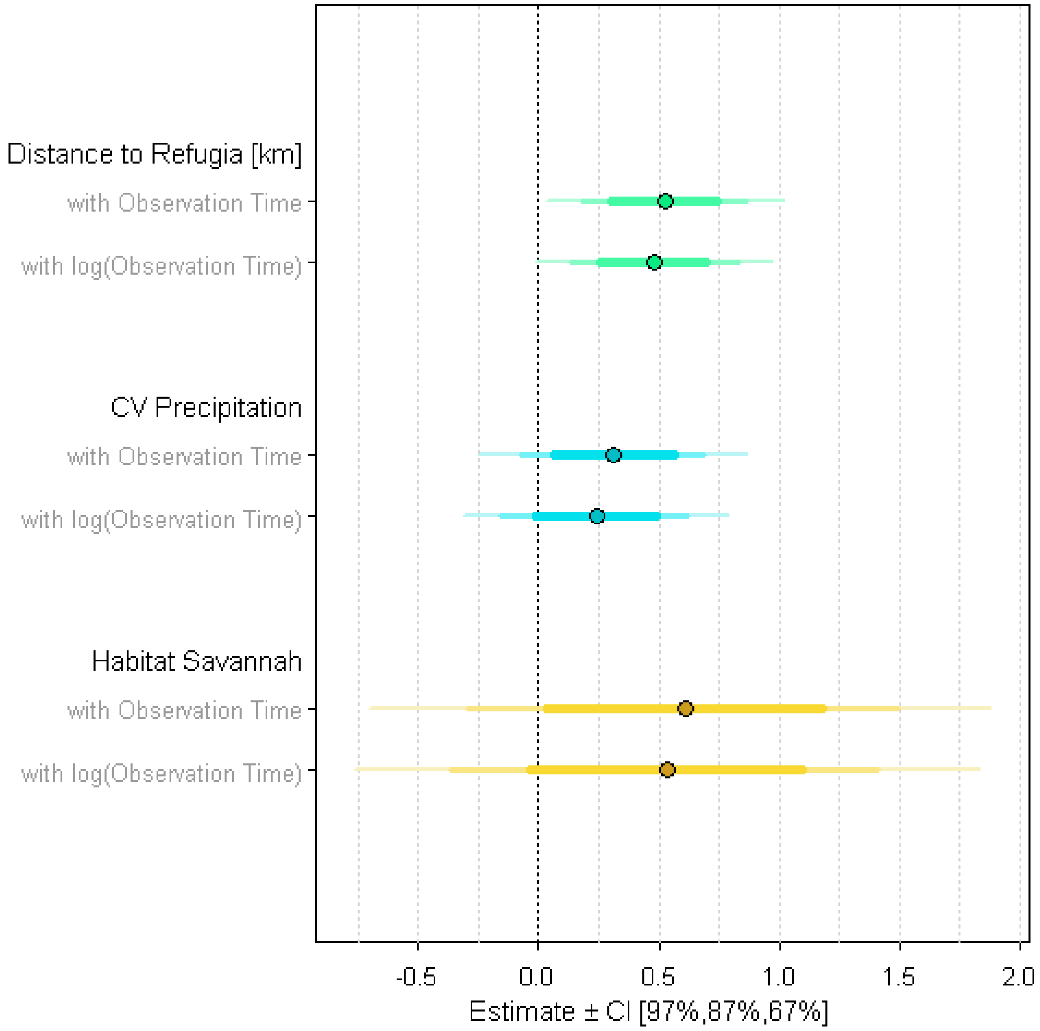

<u>Supplementary Figure 3.</u> A comparison of the positive effect of observation time untransformed versus log transformed in the models testing the effects of the three environmental variability predictors on 31 chimpanzee behaviours using a weak prior (n=144 chimpanzee communities). The plot illustrates that the treatment of observation time has little effect on the result. Shown are the means of the marginal posterior distribution (dots) and the 67%, 87%, 97% credible intervals centred on the mean (coloured areas).

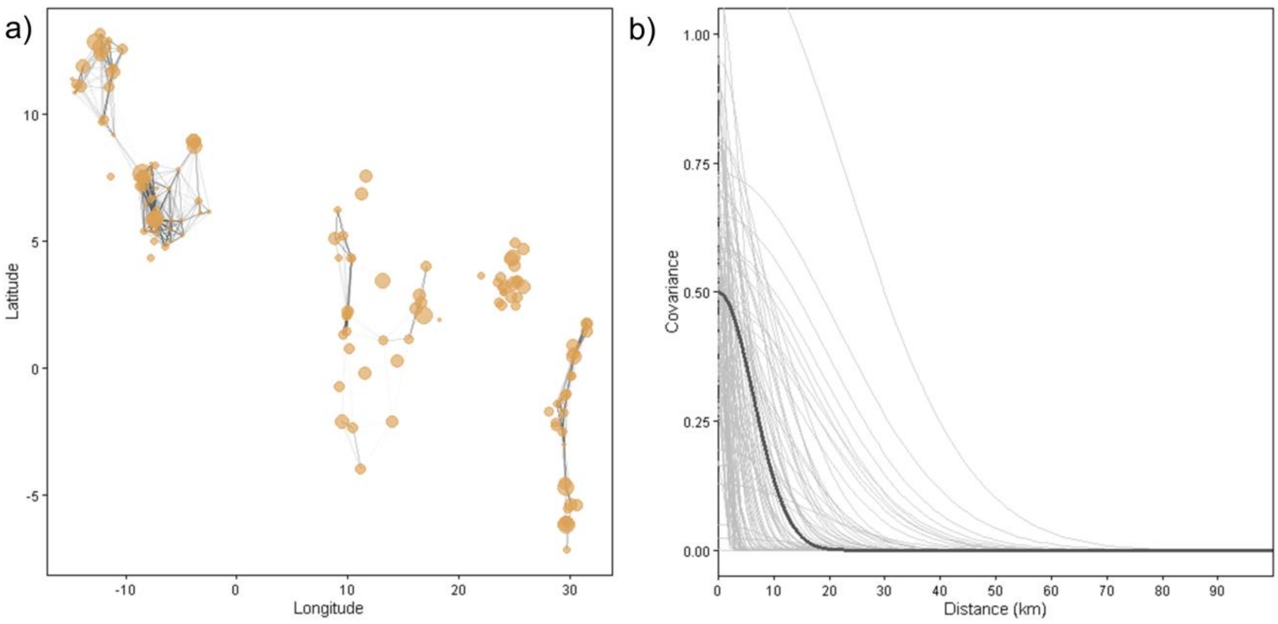

<u>Supplementary Figure 4.</u> **Spatial covariance among the 144 chimpanzee communities is evident for communities that a) cluster within close geographic coordinates, and b) declines to zero after a spatial radius, or distance, of 50-70km.** In a), the sizes of the circles represent the number of behaviours found in the chimpanzee community and thicknesses of the lines correspond to the covariance between the connected communities. In b), the black line depicts the posterior mean covariance and the thin lines show 100 functions sampled from the posterior distribution, which reveal some uncertainty about the spatial covariance in our data.
