# Supplementary material for: Environmental variability supports chimpanzee behavioural diversity
Source: Nat Commun. 2020 Sep 15;11:4451. doi: 10.1038/s41467-020-18176-3 (PMC7493986; doi:10.1038/s41467-020-18176-3)
Supplement: Supplementary file 3 — Reporting Summary [file 41467_2020_18176_MOESM3_ESM.pdf]

nature research

Corresponding author(s): Ammie K. Kalan

Last updated by author(s): July 20, 2020

Reporting Summary

Nature Research wishes to improve the reproducibility of the work that we publish. This form provides structure for consistency and transparency in reporting. For further information on Nature Research policies, seeAuthors & Referees and theEditorial Policy Checklist .

Statistics

For all statistical analyses, confirm that the following items are present in the figure legend, table legend, main text, or Methods section.

n/a	Confirmed	
☐	☒	The exact sample size (*n*) for each experimental group/condition, given as a discrete number and unit of measurement
☐	☒	A statement on whether measurements were taken from distinct samples or whether the same sample was measured repeatedly
☐	☒	The statistical test(s) used AND whether they are one- or two-sided<br>*Only common tests should be described solely by name; describe more complex techniques in the Methods section.*
☐	☒	A description of all covariates tested
☐	☒	A description of any assumptions or corrections, such as tests of normality and adjustment for multiple comparisons
☐	☒	A full description of the statistical parameters including central tendency (e.g. means) or other basic estimates (e.g. regression coefficient) AND variation (e.g. standard deviation) or associated estimates of uncertainty (e.g. confidence intervals)
☒	☐	For null hypothesis testing, the test statistic (e.g. *F*, *t*, *r*) with confidence intervals, effect sizes, degrees of freedom and *P* value noted<br>*Give P values as exact values whenever suitable.*
☐	☒	For Bayesian analysis, information on the choice of priors and Markov chain Monte Carlo settings
☒	☐	For hierarchical and complex designs, identification of the appropriate level for tests and full reporting of outcomes
☐	☒	Estimates of effect sizes (e.g. Cohen's *d*, Pearson's *r*), indicating how they were calculated

Our web collection on statistics for biologists contains articles on many of the points above.

Software and code

Policy information about availability of computer code

Data collection	no software was used to collect data
Data analysis	R version 3.5.3 and custom code for running models is included as the file Supplementary Code.

For manuscripts utilizing custom algorithms or software that are central to the research but not yet described in published literature, software must be made available to editors/reviewers. We strongly encourage code deposition in a community repository (e.g. GitHub). See the Nature Research guidelines for submitting code & software for further information.

Data

Policy information about availability of data

All manuscripts must include a data availability statement. This statement should provide the following information, where applicable:

- Accession codes, unique identifiers, or web links for publicly available datasets
- A list of figures that have associated raw data
- A description of any restrictions on data availability

The chimpanzee behavioural dataset used for this paper have been uploaded as Supplementary Data 1. All other datasets used in this study are publicly available and a list of these along with their respective links can be found in the 'Data Availability' statement.

Field-specific reporting

Please select the one below that is the best fit for your research. If you are not sure, read the appropriate sections before making your selection.

☐ Life sciences ☐ Behavioural & social sciences ☒ Ecological, evolutionary & environmental sciences

Ecological, evolutionary & environmental sciences study design

All studies must disclose on these points even when the disclosure is negative.

Study description	We updated a previously published dataset on the presence of direct or indirect evidence for 31 chimpanzee behaviours across 144 wild chimpanzee groups (also known as communities). For 46 communities data were compiled from field work carried out as part of the Pan African Programme ('PanAf') and data on these behaviours was available for 106 communities from the published literature. Since 8 groups were also sampled by the PanAf we had a total of 144 unique groups. Using a Bayesian regression analysis, we tested the effects of three environmental factors on the probability that these chimpanzee behaviours would be present in a given community. The three predictors were distance to Pleistocene forest refugia (as per the designations given by Maley 1996), precipitation seasonality (CV of precipitation) and a predominantly savanna or forest habitat. All models controlled for effects of human impact (found in a previous study), observation months per group, chimpanzee subspecies, spatial autocorrelation and included relevant random effects and slopes.
Research sample	The research includes data collected in the field on wild chimpanzee populations by the Pan African Programme (http://panafrican.eva.mpg.de) and published literature on the behaviours targeted in this study. The dataset has also been published as part of the supplementary material in a previous study (Kühl et al. 2019 Science doi:10.1126/science.aau4532) but has been updated here based on new information provided to us by people working in the field at these sites. These changes were minor (affected 8 rows of 4464). The updated dataset has also been uploaded with this manuscript as Supplementary Data 1. The general rationale behind the choice of PanAf research sites included sampling across all four subspecies, with representation of both savannah woodland and rainforest sites, and targeting locations where chimpanzees are thought to exist but the populations are not well studied by researchers (i.e., unhabituated to human observers at the time of sampling and relatively little known about these communities). In the end, the 46 sites where we were successful in collecting data relied heavily on cooperation with local NGOs, governmental authorities and local people whereas in some places it was impossible to establish a temporary research presence.
Sampling strategy	Published books and peer-reviewed studies via Google search engine were mined for relevant information on the 31 chimpanzee behaviours from the published literature (details in Kühl et al. 2019 Science doi:10.1126/science.aau4532) and all the data we collected in the field via the PanAf were included to give a total sample size of 144 unique chimpanzee communities where information on these 31 behaviours were available.
Data collection	Data collection procedure for the PanAf is available freely online at http://panafrican.eva.mpg.de/english/approaches_and_methods.php and has been briefly summarized in the methods. The data collection procedure has also been described in detail in a previous study (Kühl et al. 2019 Science doi:10.1126/science.aau4532). PanAf field sites were chosen specifically to target geographic locations within the known chimpanzee range where little prior research had been done, across all subspecies and encompassing both savanna woodland and forest environments.
Timing and spatial scale	The published literature consulted dates back to as early as 1951 all the way to the end of 2017. The PanAf data collection began in 2010 and the last site finished in 2018. The number of PanAf observation months at each chimpanzee community ranged from 1-30 months and from the published literature observation months per community range from 1-684 months, the latter including longterm research sites that have been collecting data for decades. Further details are found in our previous publication Kühl et al. 2019 Science (doi:10.1126/science.aau4532).
Data exclusions	No data were excluded because we coded both indirect and direct forms of evidence as indicative of the behaviour being present in the chimpanzee community.
Reproducibility	All data, including the updated data set and the code used to run the models in R have been uploaded as part of the supplementary files of this manuscript to aid reproducibility. The results of this study have been verified to be reproduced using the Supplementary Data 1 file and the Supplementary Code. The data set also cites all published references and/or PanAf source for the information used to code the presence of direct or indirect evidence for each chimpanzee behaviour per community.
Randomization	Randomization is not applicable since PanAf data was collected at sites particularly chosen because they included understudied or unknown chimpanzee populations.
Blinding	Not relevant since this study did not include any experiments.

Did the study involve field work? ☒ Yes ☐ No

Field work, collection and transport

Field conditions	PanAf field work was conducted in the tropical equatorial belt of Africa with high humidity of up to 99%, temperatures of up to 45 degrees Celsius and high rainfall of up to 3000 mm per year.
Location	The 46 PanAf chimpanzee communities were located across the geographic range of wild chimpanzees ranging from Senegal to Rwanda and the published literature also describes chimpanzees living across this geographic range. The PanAf field work was conducted within the following ranges of latitude/longitude: 13.183/-12.294 (northern limit), 11.925/ -13.897 (western limit), -7.129/ 29.695 (southern limit), 1.793/ 31.445 (eastern limit). The exact coordinates of all 144 chimpanzee communities included in this study (both from PanAf field work and the published literature) are in the file Supplementary Data 1.

Access and import/export	All field data collection was done with the permission of government ministries and supported by relevant host country institutions who are all named in the acknowledgments section of the manuscript. The present study does not report on any biological samples collected from the field.
Disturbance	All PanAf field research sites were temporary (1 to 30 months duration) and were removed upon completion. Clearing of bush for putting up tents and conducting surveys was kept to a minimum and no garbage or materials were left behind at field sites. Disturbance to wildlife was also minimal since all data collection methods were non-invasive.

Reporting for specific materials, systems and methods

We require information from authors about some types of materials, experimental systems and methods used in many studies. Here, indicate whether each material, system or method listed is relevant to your study. If you are not sure if a list item applies to your research, read the appropriate section before selecting a response.

Materials & experimental systems

n/a	Involved in the study	
☒	☐	Antibodies
☒	☐	Eukaryotic cell lines
☒	☐	Palaeontology
☐	☒	Animals and other organisms
☒	☐	Human research participants
☒	☐	Clinical data

Methods

n/a	Involved in the study	
☒	☐	ChIP-seq
☒	☐	Flow cytometry
☒	☐	MRI-based neuroimaging

Animals and other organisms

Policy information about studies involving animals; ARRIVE guidelines recommended for reporting animal research

Laboratory animals	No laboratory animals were involved in this study.
Wild animals	All methods for collecting data using the PanAf protocol are non-invasive and observational via infrared-sensor camera-trap devices and opportunistic observations during surveys and environmental sample collection.
Field-collected samples	No organic sample collection or lab work was used for this study.
Ethics oversight	Multiple government ministries and organizations approved and provided the PanAf with research permits to conduct field work in their countries: Ministère de la Recherche Scientifique et de l'Innovation, Ministère des Forêts et de la Faune, and the Conservation Society of Mbe Mountains, Cameroon; Ministère de la Recherche Scientifique, Ministère des Eaux et Forêts and Ministère de l'Environnement, Côte d'Ivoire; Institut Congolais pour la Conservation de la Nature and Ministère de la Recherche Scientifique, Democratic Republic of Congo; Agence Nationale des Parcs Nationaux, Centre National de la Recherche Scientifique et Technologique and Société Equatoriale d'Exploitation Forestière, Gabon; Department of Wildlife and Range Management and the Forestry Commission, Ghana; Ministère de l'Agriculture de l'Elevage et des Eaux et Forêts, Guinea; Instituto da Biodiversidade e das Áreas Protegidas and Ministro da Agricultura e Desenvolvimento Rural, Guinea-Bissau; Forestry Development Authority, Liberia; Ministre de l'Environnement et de l'Assainissement et du Developpement Durable and des Eaux et Forêts, Mali; Conservation Society of Mbe Mountains and National Park Service, Nigeria; Ministère de l'Economie Forestière, Ministère de le Recherche Scientifique et Technologique and Agence Congolaise de la Faune et des Aires Protégées, Republic of Congo; Ministry of Education and Rwanda Development Board, Rwanda; Direction des Eaux, Forêts Chasses and La Conservation des Sols, and Réserve Naturelle Communautaire de Dindéfélo, Senegal; Ministry of Agriculture, Forestry and Food Security and the National Protected Area Authority, Sierra Leone; Tanzania Commission for Science and Technology and Tanzania Wildlife Research Institute, Tanzania; Uganda National Council for Science and Technology, Uganda Wildlife Authority and Makerere University Biological Field Station, Uganda.

Note that full information on the approval of the study protocol must also be provided in the manuscript.
