# Supplementary material for: Environmental variability supports chimpanzee behavioural diversity
Source: Nat Commun. 2020 Sep 15;11:4451. doi: 10.1038/s41467-020-18176-3 (PMC7493986; doi:10.1038/s41467-020-18176-3)
Supplement: Supplementary file 4 — Description of Additional Supplementary Files [file 41467_2020_18176_MOESM4_ESM.pdf]

Description of Additional Supplementary Files

File Name: Supplementary Data 1
Description: An excel file detailing the metadata associated with all 144 chimpanzee communities and their respective coding for the 31 behaviours investigated in this study.

File Name: Supplementary Code 1
Description: The R code and model output needed to run the full model with all chimpanzee behaviours.
